# Candidemia in pediatric burn patients: Risk factors and outcomes in a retrospective cohort study

**DOI:** 10.18502/cmm.6.3.4663

**Published:** 2020-09

**Authors:** Behnam Sobouti, Mostafa Dahmardehei, Shahrzad Fallah, Majid Karrobi, Yaser Ghavami, Reza Vaghardoost

**Affiliations:** 1 Department of Pediatrics, Ali-Asghar Children Hospital, Iran University of Medical Sciences, Tehran, Iran; 2 Department of Plastic Surgery, Burn Research Center, Motahari Hospital, Iran University of Medical Sciences, Tehran, Iran; 3 Mofid Children Hospital, Shahid Beheshti University of Medical Sciences, Tehran, Iran

**Keywords:** Burn injury, Candidemia, *Candida* albicans, *C. Glabrata*, *C. Krusei*, Pediatric burn

## Abstract

**Background and Purpose:**

Despite advances in burn care and management, infections are still a major contributor to morbidity and mortality rates in patients with burn injuries. Regarding this, the present study was conducted to investigate the prevalence and importance of candidemia in pediatric burn patients.

**Materials and Methods:**

Blood samples were collected from the patients and cultured in an automated blood culture system. *Candida* species were identified using specific culture media. The relationship between candidemia and possible risk factors was evaluated and compared to a control group.

**Results:**

A total of 71 patients with the mean age of 4.52±3.63 years were included in the study. Blood cultures showed candidemia in 19 (27%) patients. Based on the results, *C. albicans* was the most common fungus among patients with and without candidemia. The results of statistical analysis also showed that candidemia was significantly correlated with total body surface area (TBSA), mechanical ventilation, duration of total parenteral nutrition, length of intensive care unit (ICU) stay, presence of neutropenia, and R-Baux score (all *P≤0.001*). In this regard, TBSA, length of ICU stay, R-Baux score, and *Candida* score were identified as the determinant factors for mortality due to candidemia.

**Conclusion:**

Candidemia increases the mortality and morbidity rates associated with burn injuries. Prompt diagnostic and prevention measures can reduce the unfortunate outcomes via controlling the possible risk factors.

## Introduction

Septicemia and infections are the major causes of death in burn patients [ 1 ], with bacteria being the main microorganisms isolated from these cases [ 2 , 3 ]. However, other pathogens, such as fungal species, are significantly detected in burn wound infections [ 4 ]. *Candida* species are reported to be responsible for approximately 3-23% of all bloodstream infections with a variable mortality rate, which might reach up to 71% [ 5 - 9 ]. In most cases, wound colonization can eventually lead to systemic infection [ 3 ]. Candidiasis in burn patients is associated with high rates of mortality and morbidity and long hospital stay [ 10 , 11 ].

Necrotic tissue provides an ideal environment for the colonization and growth of different microorganisms, such as bacteria and fungi [ 4 ]. Other risk factors predisposing the burn patients to fungal infections include broad-spectrum antibiotics, corticosteroids treatment, mechanical ventilation, total parenteral nutrition (TPN), tracheostomy tubes, hyperglycemia caused by burn injuries, renal failure, consistent neutropenia, and immune system dysfunction [ 9 , 12 - 15 ].

Candida infections impose high costs on the healthcare system [ 16 ]. In an analysis performed around two decades ago, the cost of candidemia in adults was estimated at about $34,000-45,000, which was mostly due to the cost of prolonged hospital stay [ 16 , 17 ]. In another study investigating candidemia in children, the average hospital cost was about $184,000 with a mean stay length of 45 days and a high mortality rate of 16% [ 8 ]. The rates reported for mortality due to candidemia in burn patients range from 10% to 90% in different studies [ 8 - 10 , 18 - 21 ].

In previous studies, *C. albicans* was considered the major cause of candidiasis in burn patients [ 13 , 22 - 27 ].

In recent years, infection with non-albicans *Candida* species has been emerging [ 9 , 28 , 29 ]. It has been reported that non-albicans *Candida* species have lower sensitivity to the usual antifungal drugs used for the treatment of *C. albicans* infections [ 18 , 29 - 32 ]. This issue can raise a major concern in the treatment of burn patients with fungal infections caused by non-*albicans Candida* species. With this background in mind, the current study was conducted to investigate the presence of candidemia and different *Candida* species in pediatric patients with burn injuries.

## Materials and Methods

This retrospective cohort study was conducted in Shahid Motahari Burn Hospital, Tehran, Iran, from January 2017 to February 2019. A total of 327 medical records of pediatric patients aged younger than 18 years who were hospitalized due to burn injuries were studied. Patients with any type of *Candida* infection were enrolled in this study. This research was approved by the Ethics Committee of Iran University of Medical Sciences (ethical code: IR.IUMS.FMD.REC.1398.273). Informed consent for participation in the study and access to medical records was obtained from the parents or guardians of the patients.

Demographic data, such as age, gender, total body surface area (TBSA), mechanism of burn injury, use of mechanical ventilation, presence of neutropenia, and inhalational injuries, were recorded using the patients' medical records. Based on the data of the medical records, all patients had been evaluated at the emergency department and received proper resuscitation by intravenous fluids using the Parkland formula. Considering the high incidence of fungal infections in burn patients after the second week of burn injuries, the samples were collected 2 weeks after patient admission to the burn treatment center.

**Inclusion Criteria**

Patients with the TBSA burn of > 30%, consistent fever despite antibiotic therapy for more than 4 days, and evidence of wound discoloration was tested for fungal infections.

**Fungal Identification**

Sample Collection: Based on patient medical records, sample collection was carried out using the following process. A total of 184 samples from different suspicious sites, such as wound, urine, oral and respiratory secretions, and central venous catheter (CVC), were collected from the patients using sterile swabs. In patients with CVC, 10 mL blood was drawn through CVC line and sent to the laboratory in a sterilized bottle. For patients without CVC line, the skin was sterilized using 70% alcohol swabs, and 10 mL blood was collected from the cubital fossa or another accessible location on the arm or hand and then placed into sterilized bottles. Furthermore, oral and respiratory sampling was accomplished using a sterilized swab inserted into the mouth or in some patients, into the tracheostomy tubes. The swabs were then placed in a sterilized bottle and sent to the laboratory. Urine samples were also obtained through indwelling catheters using a sterile syringe; 10 mL urine was collected and sent to the laboratory in sterile bottles.

Sample Culture: Based on the patients’ medical records, all samples (i.e., blood samples and swabs from the urine, wound, and trachea) were cultured. In doing so, the urine samples were cultured using calibrated loop onto either chromogenic clear media (Oxoid Ltd, Basingstoke, UK) or CHROMID *Candida* agar (Biomerieux, France). A positive culture was defined as ≥ 10^5^ CFU/ml. Blood samples were cultured using BATEC 9240 blood culture system in Aerobic Plus/F bottles. This system uses infrared spectrophotometry to monitor carbon dioxide produced by microorganisms on a continuous basis. The subcultures were performed on both Sabouraud glucose agar (Merck, Darmstadt, Germany) and CHROM agar (Merck, Darmstadt, Germany). The CHROM agar media enables the selective isolation and differentiation of *Candida* species based on the colony color and morphology.

Culture is still the gold standard method for the detection of candidiasis [ 33 ]. The high sensitivity and specificity of this method has been described in previous studies for the detection of the most commonly encountered *Candida* species [ 34 , 35 ]. Both plates were incubated at 37°C for 48-72 h. The chromatic characteristics of the colonies included light to medium green colonies identified as *C. albicans*, dark pink to purple colonies detected as *C. glabrata*, steel blue colonies identified as *C. tropicalis*, and pink with whitish border colonies (rose-colored) described as *C. krusei*. All isolates were confirmed by the API 20 C AUX strip (BioMerieux, Marcy l’Etoile, France). *Candida albicans* isolates were confirmed by their ability to grow at 45°C. Frequency of *Candida* species in patients is depicted in Table 1.

**Table 1:** Frequency of *Candida* species in patients with and without candidemia and survivor and deceased patients from candidemia

*Candida* species (%)	Patients without candidemia (n=52)	Patients with candidemia (n=19)	Deceased patients with candidemia (n=11)	Survived patients with candidemia (n=8)	All patients (n=71)
*C. albicans*	23 (44.2)	8 (42.1)	3 (27.3)	5 (62.5)	31 (43.7)
*C. krusei*	1 (1.9)	4 (21.1)	4 (36.4)	0	5 (7)
*C. parapsilosis*	9 (17.3)	4 (21.1)	1 (9.1)	3 (37.5)	13 (18.3)
*C. glabrata*	7 (13.5)	3 (15.8)	3 (27.3)	0	10 (14.1)
*C. tropicalis*	12 (23.1)	0	0	0	12 (16.9)

*Candida* species were detected using BACTEC 9240 culture system.

**Susceptibility Testing**

Antifungal susceptibility was performed using the E-test (bioMerieux, France) strip tests according to the instructions provided by the manufacturer [ 36 ]. The E-test strips of itraconazole (0.002 ∼ 32 μg/mL), fluconazole (0.016 ∼ 256 μg/mL), and amphotericin B (0.002 ∼ 32 μg/mL) were used in the study. Interpretive susceptibility criteria for antifungal breakpoints were adapted from the Clinical and Laboratory Standards Institute. Antifungal treatment with amphotericin B, fluconazole, and itraconazole was started for the patients with *Candida* infections based on antifungal susceptibility testing (Table 2).

**Table 2:** Susceptibility results for isolated *Candida* species

*Candida* Species	Antifungal agent	Average MIC (µg/ml) using Etest
*C. albicans* (n=8)	Itraconazole	0.004~0.095
	Fluconazole	0.065~0.77
	Amphotericin B	0.015~0.21
*C. glabrata* (n=3)	Itraconazole	0.24~3.2
	Fluconazole	Resistant
	Amphotericin B	0.32~1.2
*C. parapsilosis* (n=4)	Itraconazole	0.023~0.14
	Fluconazole	2~6
	Amphotericin B	0.14~0.22
*C. krusei* (n=4)	Itraconazole	0.22~3.4
	Fluconazole	26~65
	Amphotericin B	0.1~2.2

MIC: minimum inhibitory concentration

* Minimum inhibitory concentration at 24-48 h measurement

**Score Calculation**

Revised-Baux (R-Baux) score was calculated using the following formula to measure the prognosis of the patients:

R-Baux Score=TBSA+Patient’s age+17 (inhalation injury, 1=yes, 0=no)

*Candida* score was calculated in all patients in order to decide which patient will benefit from early antifungal treatment. A *Candida* score of > 3 was considered positive for the initiation of antifungal treatment.

*Candida* Score=Severe sepsis (2 points)+TPN (1 point)+surgery (1 point)+multifocal *Candida* colonization (1 point)

**Statistical Analysis**

The collected data included the duration of broad- spectrum antibiotics consumption, severe sepsis, TPN, depth of burns, mechanical ventilation, presence of neutropenia, presence of inhalational injury, presence of central venous catheters, and presence of underlying diseases. The data were reported as mean±standard deviation and percentage. The comparison of the continuous and categorical variables was performed using the t-test and Chi-square test, respectively. In addition, ANOVA test was used for comparing multiple variables. The measurement of survival was also accomplished using Kaplan-Meyer test. Statistical analyses were performed in SPSS software for Windows (version 21, Armonk, NY:IBM Corp.). The level of significance was considered < 0.05, and 95% confidence interval was applied.

## Results

**Figure 1:**
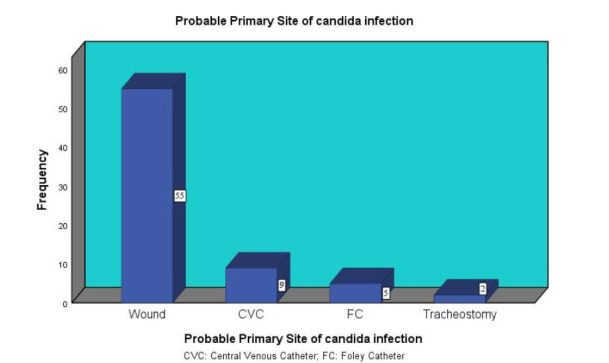
Prevalence of *Candida* species in different sites in all patients

**Figure 2:**
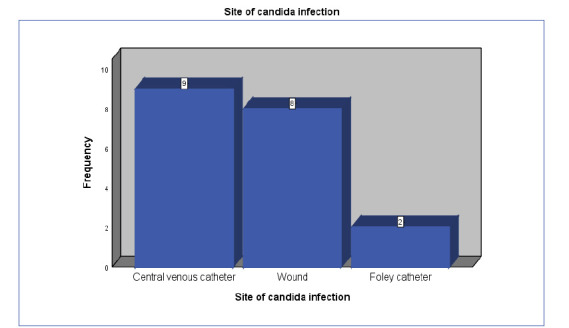
Prevalence of *Candida* species in different sites in patients with candidemia

A total of 71 patients with *Candida* infection in different body sites were included in this study. Out of this population, 19 patients had positive blood culture for *Candida* species (19/71, 27%). Demographic data, TBSA, and mechanism of burn injuries are shown in Table 3. All patients with *Candida* infections (with or without candidemia) received intravenous antibiotics. In addition, all patients with candidemia were subjected to TPN. The prevalence of *Candida* in different sites is depicted in Figure 1. Culture results related to the recovery of *Candida* species in patients with candidemia is illustrated in Figure 2.

**Table 3:** Demographic variables, degree, and burn percentage, mechanisms of burns, and other risk factors for patients with and without candidemia

Characteristic	Patients with candidemia (n=19)	Patients without candidemia (n=52)	Total (n=71)
Age (year) Mean±SD	4.79±4.01	4.38±3.51	4.52±3.63
Gender (%)	M: 10 (52.6)	M: 31 (59.6)	M: 41 (57.7)
	F: 9 (47.4)	F: 21 (40.4)	F: 30 (42.3)
Deceased (%)	11 (58)	17 (32)	28 (39)
TBSA% Mean±SD	51.79±12.84	35.1±12.82	39.56±14.75
Degree of burn			
II	0	15 (28.8)	15 (21.1)
III	6 (31.6)	14 (26.9)	20 (28.2)
IV	4 (21.1)	0	4 (5.6)
II & III	3 (15.8)	22 (42.3)	25 (35.2)
III & IV	6 (31.6)	1 (1.9)	7 (9.9)
Percentage of burn area 15-30% (%)			
15-30% (%)	0	22 (42.3)	22 (31)
31-50% (%)	10 (52.6)	23 (44.2)	33 (46.5)
>50% (%)	9 (47.4)	7 (13.5)	16 (22.5)
Mechanism of burn (%)			
Hot liquids	9 (47.4)	28 (53.8)	37 (52)
Gas explosion	9 (47.4)	22 (42.3)	31 (43)
Electrical injury	1 (5.3)	1 (1.9)	2 (2.8)
Chemical	0	1 (1.9)	1 (1.4)
Use of mechanical ventilation	12 (63.2)	20 (38.5)	32 (45.1)
Presence of neutropenia (%)	15 (78.9)	16 (31)	31 (43.7)
Presence of inhalational injury (%)	5 (26.3)	10 (19.2)	15 (21.1)

TBSA: total body surface area

Based on the results of the data analysis, candidemia had a significant correlation with TBSA, duration of TPN, burn degree, duration of antifungal treatment, length of ICU stay, R-Baux score, *Candida* score, and presence of neutropenia as compared to the control group (Table 4). However, no correlation was observed between candidemia and the duration of antibiotic treatment (*p*=0.07).

**Table 4:** Comparison of the risk factors for patients with and without candidemia

Variable	Patients with candidemia (n=19)	Patients without candidemia (n=52)	P-value
TBSA%	51.79±12.84	35.1±12.82	0.001
Duration of mechanical ventilation (days)	6.63±6.15	0.8±1.35	0.001
Duration of TPN (days)	17.37±3.06	7.06±6.74	0.001
Duration of antibiotic therapy (days)	22.21±3.55	25.52±7.49	0.07
Time frame between burn injury and start of antifungal therapy (days)	8.89±1.59	10.94±2.94	0.005
Duration of antifungal therapy (days)	12.53±4.88	15.85±6.01	0.03
Length of ICU stay	28.42±10.95	5.13±6.28	0.001
R-Baux score	61.05±16.17	42.73±14.23	0.001
*Candida* score	3.42±1.17	2.69±1.21	0.02
Presence of inhalation injury	0.26 ±0.43	0.19±0.39	0.52
Presence of neutropenia (%)	0.79±0.42	0.31±0.47	0.001

TBSA: total body surface area, TPN: total parenteral nutrition, ICU: intensive care unit

Table 5 presents data related to demographic information, burn percentage and degree, mechanisms of burns, and risk factors for survived and deceased patients with candidemia. Table 6 shows the result of comparing different variables between survivors and deceased patients who had candidemia. Our findings showed a statistically significant difference between the two groups in terms of TBSA, duration of mechanical ventilation, length of ICU stay, and *Candida* score. R- Baux score showed a meaningful statistical correlation with patient mortality (*P*=0.008). Regression analysis was performed to determine the correlation between candidemia and possible risk factors in the study (Table 7). Furthermore, the relationship between mortality rate and other risk factors in all patients was analyzed using linear regression test (Table 8).

**Table 5:** Demographic variables, burn degree and percentage, mechanisms of burns, and risk factors for survived and deceased patients with candidemia

Characteristic	Survived from candidemia (n=8)	Deceased from candidemia (n=11)	Total (n=19)
Age (year) Mean ±SD	4.88±3.56	5.09±5.19	4.52±3.63
Gender (%)	M: 5 (62.5)	M: 5 (45.5)	M: 10 (52.6)
	F: 3 (37.5)	F: 6 (54.5)	F: 9 (47.4)
TBSA% Mean±SD	44.88±12.69	56.82±10.87	39.56±14.75
Degree of Burn			
III	4 (50)	2 (18.2)	6 (31.6)
IV	0	4 (36.4)	4 (21.1)
II & III	2 (25)	1 (9.1)	3 (15.8)
III & IV	2 (25)	4 (36.4)	6 (31.6)
Percentage of burn area			
31-50% (%)	7 (87.5)	3(27.3)	10 (52.6)
>50% (%)	1 (12.5)	8 (72.7)	9 (47.4)
Mechanism of burn (%)			
Hot liquids	4 (50)	5 (45.5)	9 (47.4)
Gas explosion	3 (37.5)	6 (54.5)	9 (47.4)
Electrical injury	1 (12.5)	0	1(5.3)
Chemical	0	0	0
Use of mechanical ventilation	1 (12.5)	11 (100)	12 (63.2)
Presence of neutropenia (%)	6 (75)	9 (81.8)	15(78.9)
Presence of inhalational injury (%)	1 (12.5)	4 (36.4)	5 (26.3)

**Table 6:** Comparison of risk factors between survivors and deceased patients with candidemia

Variable	Survived (n=8)	Deceased (n=11)	P-value
TBSA%	44.88±12.69	56.82±10.87	0.04
Duration of mechanical ventilation (days)	1.00±2.83	10.73±4.34	0.001
Duration of TPN (days)	16.88±2.42	17.73±3.52	0.56
Duration of antibiotic therapy (days)	22.25±3.88	22.18±3.49	0.97
Time interval between burn injury and onset of antifungal therapy (days)	8.5±1.60	11.73±4.86	0.37
Duration of antifungal therapy (days)	13.63±5.01	11.73±4.86	0.42
Length of ICU stay	21.38±6.55	33.55±10.83	0.012
R-Baux score	52.00±13.54	67.64±15.14	0.03
*Candida* score	2.25±0.46	4.27±0.65	0.001
Presence of neutropenia (%)	0.75±0.46	0.82±0.41	0.74
Presence of inhalational injury	0.13±0.35	0.36±.51	0.27

TBSA: total body surface area, TPN: total parenteral nutrition , ICU: intensive care unit

**Table 7:** Factors associated with positive *Candida* blood culture (candidemia) using linear regression test

Model	Coefficients^a^						
	Unstandardized coefficients		Standardized coefficients			95.0% Confidence interval for B	
	B	Std. Error	Beta	T	Sig.	Lower bound	Upper bound
(Constant)	-1.863	0.710		-2.625	0.011	-3.286	-0.440
Site of *Candida* infection	0.309	0.160	0.151	1.935	0.058	-0.011	0.629
Patient age	-0.080	0.053	-0.195	-1.526	0.133	-0.186	0.025
Patient gender	-0.135	0.239	-0.044	-0.565	0.575	-0.615	0.345
Percentage of burn area	-0.072	0.556	-0.035	-0.129	0.898	-1.187	1.043
Degree of burn	-0.051	0.094	-0.046	-0.546	0.587	-0.240	0.137
Use of mechanical ventilation	-0.602	0.315	-0.198	-1.909	0.062	-1.235	0.030
Mechanism of burn	0.444	0.233	0.184	1.907	0.062	-0.023	0.911
Presence of inhalation injury	-0.674	0.542	-0.179	-1.242	0.219	-1.762	0.414
r baux score	0.018	0.028	0.199	0.633	.529	-0.039	0.074
Duration of mechanical ventilation	0.138	0.051	0.385	2.710	.009	0.036	0.239
Total parenteral nutrition	0.218	0.343	0.051	0.635	.528	-0.469	0.904
Length of ICU stay (days)	0.037	0.013	0.312	2.744	.008	0.010	0.063
Presence of neutropenia	-0.011	0.269	-0.004	-0.040	.968	-0.551	0.529

a. Dependent variable: blood culture *Candida* species (candidemia)

**Table 8:** Factors associated with higher mortality rate using linear regression test

Model	Coefficients^a^						
	Unstandardized coefficients		Standardized coefficients			95.0% Confidence interval for B	
	B	Std. Error	Beta	T	Sig.	Lower bound	Upper bound
(Constant)	0.554	0.199		2.779	0.008	0.153	0.955
Patient age	0.046	0.034	0.350	1.370	0.177	-0.022	0.115
Patient gender	-0.013	0.048	-0.014	-0.282	0.779	-0.109	0.082
Percentage of burn area	-0.034	0.102	-0.050	-0.331	0.742	-0.239	0.171
Degree of burn	0.013	0.017	0.037	0.777	0.441	-0.021	0.048
Use of mechanical ventilation	0.450	0.088	0.458	5.098	0.000	0.272	0.627
Mechanism of burn	0.010	0.043	0.013	0.236	0.815	-0.077	0.097
Presence of inhalation injury	0.694	0.611	0.571	1.135	0.262	-0.536	1.923
R Baux score	-0.045	0.036	-1.564	-1.260	0.214	-0.117	0.027
Time of mechanical ventilation	0.005	0.010	0.039	0.458	0.649	-0.015	0.024
Using total parenteral nutrition	0.118	0.079	0.085	1.498	0.141	-0.040	0.276
Total parenteral nutrition time (days)	-0.004	0.005	-0.062	-0.758	0.452	-0.015	0.007
Beginning time of antibiotic	0.023	0.055	0.033	0.421	0.675	-0.088	0.134
Total duration of antibiotic treatment (days)	0.005	0.007	0.073	0.770	0.445	-0.009	0.019
Onset time of antifungal treatment	-0.014	0.011	-0.080	-1.283	0.206	-0.036	0.008
Total duration of antifungal treatment (days)	0.004	0.006	0.047	0.594	0.555	-0.009	0.017
Presence of neutropenia	0.092	0.049	0.093	1.881	0.066	-0.006	0.190
Length of ICU stay (days)	0.007	0.004	0.194	1.802	0.078	-0.001	0.016
Length of hospital stay	-0.006	0.005	-0.105	-1.255	0.216	-0.015	0.003
*Candida* score	0.177	0.029	0.449	5.988	0.000	0.117	0.236

## Discussion

In the present study, *C. albicans* was identified as the most common cause of fungal infection in the investigated patients, followed by *C. tropicalis*, *C. parapsilosis*, *C. glabrata*, and *C. krusei*. However, with regard to the patients with candidemia, *C. albicans* was the most common cause, followed by *C. krusei*, *C. parapsilosis*, and *C. glabrata*. There was no candidemic patient with *C. tropicalis*. Other previous studies also reported *C. albicans* as the most prevalent fungal infection in comparison to other fungal species [ 12 - 14 , 25 , 37 ]. However, in a study performed by Luo, *C. tropicalis* was reported as the dominant species in burn patients [ 9 ].

Moreover, our findings showed that *C. albicans* was more prevalent in candidemic burn patients. In a study performed by Lotfi et al. in Iran, *C. parapsilosis* was identified as the dominant pathogen in patients with burn injuries [ 28 ]. In a study carried out in China, Zhou et al. reported C. paraspsilosis as the dominant fungi recovered from candidemic burn patients [ 38 ]. The pattern of fungal infections in our cases was single-species infection, which means that only one type of *Candida* species was isolated from each infected patient. Although there are some other studies reporting mixed *Candida* infections [ 27 , 39 , 40 ], there was no case of mixed *Candida* infection and/or candidemia in the present study.

The incidence rate of candidiasis in our study was 21%, while the incidence rate of candidemia was 5.8%. These incidence rates are within the range of the values reported in other studies [ 12 , 18 , 24 , 27 , 28 ]. However, there are also some studies reporting higher incidence rates for candidemia in burn patients. Sheridan reported a candidemia incidence rate of 14.4% in pediatric burn patients [ 21 ]. Furthermore, in a study performed by Zhou, this rate was reported to be between 6.06% and 17.54% in burn patients during a 6-year period [ 38 ]. In the current study, the mortality rate was obtained as 39%. Moreover, the candidemic burn patients were found to have an increased risk of fatality [Table 1] (58%) . These findings are in accordance with those reported in previous studies [ 10 , 13 , 14 , 27 ]. However, our findings are inconsistent with those obtained by Vinsonneau et al., reporting that candidemia did not increase the mortality rate in candidemic patients in comparison to that in the control group [ 41 ].

In the current research, burn wound was the major site of fungal colonization, followed by CVC, Foley catheter, and tracheostomy tubes. In the same vein, other studies have mentioned wounds as the most common site for fungal infections in burn patients [ 11 , 18 , 19 , 21 ]. However, our findings revealed CVC as the most likely primary site of candidemia in burn patients. Central venous catheters have been implicated to account for bloodstream infections and candidemia in other studies [ 27 , 40 - 44 ]. In a study, catheter-related bloodstream infection was higher in patients with longer use of catheters and TPN, which is in agreement with other investigations. This can be due to the formation of *Candida* biofilms on the surface of catheters due to the longer use [ 45 ]. A study performed by Escrig et al. demonstrated CVC as the main source of candidemia in burn patients [ 27 ].

The duration of mechanical ventilation was longer in patients with candidemia in comparison to that in the control group (Table 3). Based on the literature, prolonged mechanical ventilation predisposes critically ill patients, including burn patients, to secondary fungal infections [ 18 , 43 , 46 , 47 ]. In addition, patients with sepsis and/or candidemia need more intensive care and ventilation supports, which can lead to a vicious cycle in the treatment of these patients [ 9 , 13 ]. In the present research, the candidemic patients had longer duration of TPN in comparison to the control group (Table 3). Previous studies have indicated a correlation between TPN and increased risk of candidemia [ 13 , 27 ]. *Candida* species are residing in the gastrointestinal tract as a normal microbiome. The translocation of these species through the intestinal mucosa is the most common pathway for *Candida* bloodstream infections.

Patients receiving TPN have been shown to have increased *Candida* biofilm production, which leads to higher virulence [ 48 ]. In addition, mucosal damage through pre-existing or iatrogenic gastric ulcers facilitates fungal entrance to the bloodstream [ 49 ]. However, a study on 60 patients with extensive burns indicated that early post-burn enteral nutrition reduced the risk of post-burn infections and duration of antibiotic treatment [ 50 ].

The use of broad-spectrum antibiotics is another risk factor that predisposes burn patients to invasive fungal infections. In our study, all patients received antibiotics for a long period. However, the duration of antibiotic treatment did not show any statistically significant difference between the case and control groups (Table 3). The interval between burn injury and onset of intravenous antifungal therapy was another variable that was shorter in the candidemic patients. Risk factors, such as TBSA of > 50%, full-thickness burn area, and moderate to severe inhalation injury, were enhanced in case of the prophylactic and early initiation of antifungal treatment in this group of patients [ 47 ]. Patients with candidemia had a shorter antifungal treatment course. This can be explained by the higher mortality rate in this group (11/19, 58%) that ended up in shorter treatment and follow-up.

**Figure 3:**
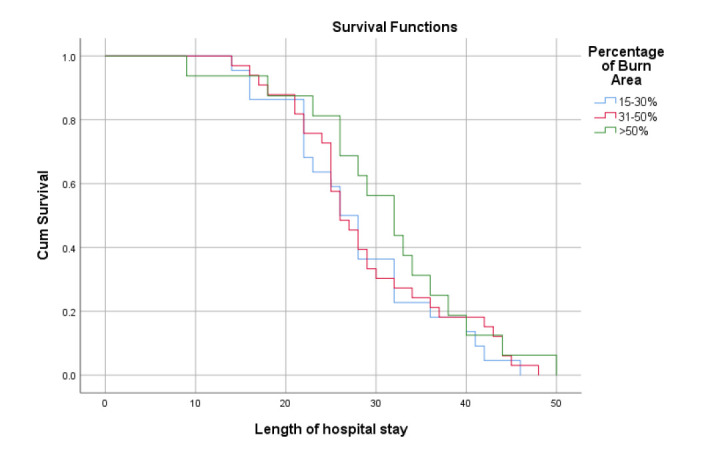
Survival analysis of patients based on total body surface area, length of hospital stay, and survival status

The TBSA is a known risk factor for predicting mortality in patients with burn injuries [4, 10, 11, 13, 14, 18, 20]. Patients with higher TBSA are more susceptible to the consequences of the burn injuries [ 4 , 8 - 14 , 17 - 21 , 51 ]. Moreover, the mortality rate is higher in the patients with higher TBSA and concomitant inhalational injuries [ 51 - 55 ]. R-Baux score is a variable that takes TBSA, patient age, and inhalational injury into consideration. This score is used to predict the prognosis and risk of mortality in patients with burn injuries [ 56 - 58 ]. In our study, both TBSA and R-Baux score were higher in patients with candidemia in comparison to those in the control group (Table 3). These two variables were also higher in deceased patients with candidemia in comparison to those in the survived patients (Table 5). The survival analysis of patients based on their TBSA showed that patients with a higher percentage of burn surface had a lower chance of survival (Figure 3).

Moreover, *Candida* score was higher in patients with candidemia, compared to that in the control group (Table 3). This score was also higher in deceased candidemic patients (Table 5); accordingly, it can be used as a predictor and a risk factor for mortality in burn patients. Our study showed that a score higher than 4 can predict a poor prognosis. In other studies, *Candida* score has been used to differentiate among ICU patients with severe sepsis or septic shock who might benefit from early antifungal treatment (score>3) [ 59 - 61 ].

Epidemiological studies in Iran show that candidemia is a growing issue in hospitalized patients [ 28 , 62 ]. The growing number of nosocomial candidiasis, especially in pediatric population, raises a major concern in considering preventative and therapeutic measures for decreasing the associated morbidities and mortalities [ 28 , 62 - 64 ]. It is noteworthy to mention that our study was a retrospective evaluation of candidemic burn patients. It was attempted to figure out the methods applied for the detection of *Candida* infections in most cases. However, most of the medical records did not have adequate information on *Candida* detection methods, and we could only report the diagnosis of candidiasis or candidemia for those cases. It is suggested to perform further studies using a wider study population to cover both adult and pediatric populations. Limitations with case-control studies, such as recall bias and lack of adequate follow-ups, apply to this study. However, we could find the prevalence of candidemia and its associated risk factors in our study population with burn injuries.

## Conclusion

As the findings indicated, the incidence of candidemia in ICU burn patients aged below 14 years was 16.3%. *Candida albicans* was the most common fungus isolated from the patients with and without candidemia. Based on the results, TBSA, inhalational injury, presence of neutropenia alongside long duration of mechanical ventilation and TPN predispose the burn patients to fungal infections.

.
